# Clinical, morphological and genetic characteristics of patients with concurrent presence of *JAK2* V617F and *BCR::ABL1*

**DOI:** 10.1038/s41598-025-11096-6

**Published:** 2025-07-18

**Authors:** Nicole Naumann, Vito Dangelo, Johannes Lübke, Jakob Bresser, Volker Hagen, Jolanta Dengler, Georgia Metzgeroth, Sebastian Kreil, Tabea Hockenberger, Wolf-Karsten Hofmann, Alice Fabarius, Susanne Saussele, Nicholas C. P. Cross, Andreas Reiter, Juliana Schwaab

**Affiliations:** 1 Department of Hematology and Oncology, University Hospital Mannheim, Heidelberg University, Mannheim, Germany; 2 Klinik für Innere Medizin II, St. Johannes Hospital Dortmund, Dortmund, Germany; 3 Onkologikum Stuttgart, Stuttgart, Germany; 4 Faculty of Medicine, University of Southampton, Southampton, UK; 5 Wessex Genomics Laboratory Service, Salisbury District Hospital, Salisbury, UK; 6 Department of Hematology and Oncology, University Hospital Mannheim Heidelberg University, Theodor-Kutzer-Ufer 1-3, 68167 Mannheim, Germany

**Keywords:** Genetics research, Myeloproliferative disease

## Abstract

Diagnosis and treatment of chronic myeloid neoplasms with two concurrently present driver mutations is challenging. We report on 10 *JAK2* V617F^pos^/*BCR::ABL1*^pos^ patients in whom both mutations were identified simultaneously in 5/10 (50%) patients or in whom *BCR::ABL1* appeared a median of 14 years after the primary diagnosis of *JAK2* V617F^pos^ myeloproliferative neoplasia (MPN) in the remaining 5 patients. Granulocyte-macrophage colony-forming unit (CFU-GM) analysis demonstrated subsequent acquisition of *BCR::ABL1* in a pre-existing *JAK2* V617F^pos^ clone in 8/9 (89%) of evaluable patients. Despite the presence of *JAK2* V617F in all patients, atypical *BCR::ABL1* transcripts (e1a2/e19a2) in 3/9 (33%) patients and additional somatic mutations in 5/9 (56%) patients, molecular remission of *BCR::ABL1* was achieved with different ABL1 TKIs (imatinib, *n* = 2, dasatinib, *n* = 2, nilotinib, *n* = 3) in 7/9 (78%) patients. During a total of 217 months of treatment, concomitant treatment with ABL1 TKIs and ruxolitinib did not affect dosing, efficacy or side effects. We conclude that (i) a second driver mutation might occur in chronic phase MPNs, (ii) clonality analyses largely support a common disease origin, and (iii) the dose, efficacy and safety of ABL1 inhibitors and ruxolitinib are not mutually affected by concurrent treatment.

**Supplementary Information:**

The online version contains supplementary material available at 10.1038/s41598-025-11096-6.

## Introduction

The World Health Organisation (WHO) and the International Consensus Classification (ICC) classify myeloproliferative neoplasms (MPN) into chronic myeloid leukemia (CML), with the characteristic presence of a *BCR::ABL1* fusion gene (*BCR::ABL1*^pos^), and *BCR::ABL1* negative (*BCR::ABL1*^neg^) disorders (i.e. essential thrombocythemia [ET], polycythemia vera [PV] and (primary/secondary/pre-fibrotic) myelofibrosis [MF]), which are variably positive for mutations in *JAK2* (*JAK2* V617F, *JAK2* V617F^pos^), *CALR* or *MPL* in > 90% of patients, commonly leading to activation of the JAK/STAT pathway. Based on genetic conditions, *BCR::ABL1*^pos^ CML and *BCR::ABL1*^neg^ MPN significantly vary on clinical phenotype, therapeutic options, response to treatment and prognosis^1,2^.

In CML, treatment with ABL1 tyrosine kinase inhibitors (ABL1-TKI) and more recently inhibitors specifically targeting the ABL myristoyl pocket (STAMP) usually results in complete hematologic, deep, and durable molecular remissions conferring into excellent long-term overall survival (OS)^3–5^. In *BCR::ABL1*^neg^ MPN, treatment with JAK1/2-inhibitors (all of which are approved for symptomatic myelofibrosis), e.g. ruxolitinib, fedratinib, momelotinib or pacritinib, heterogeneously affect splenomegaly, clinical symptoms and blood values e.g. anemia but durable hematologic and molecular remissions only rarely occur^6–8^.

In general, the concurrent presence of *JAK2* V617F and *BCR::ABL1* in the same patient is considered as extremely rare, only several case reports, small case series or reviews have reported on this phenomenon in 142 patients^9^. However, the clonal structure and assessment of response to treatment in relation to mutational profiles is reported in only a small minority of these cases. The present manuscript therefore includes both clinical data and clonality analyses (colony-forming unit granulocyte-macrophage [CFU-GM] assay) to gain a deeper insight into the clonal architecture of this rare disease.

### Patients and methods

#### Patients and samples

Overall, 10 patients with concurrent presence of *BCR::ABL1* and *JAK2* V617F (*JAK2* V617F^pos^/*BCR::ABL1*^pos^) were diagnosed within the routine diagnostic work- up in our center between 2009 and 2023. The diagnoses of *BCR::ABL1*^neg^ MPN and *BCR::ABL1*^pos^ CML were established according to the WHO classification^1^. All patients with concurrent presence of *BCR::ABL1* and *JAK2* V617F (*JAK2* V617F^pos^/*BCR::ABL1*^pos^) gave written informed consent to be included in the German Registry of Eosinophils, Mast Cells and rare myeloid neoplasms. The study design adhered to the tenets of the Declaration of Helsinki and was approved by the relevant institutional review board (Medical Faculty Mannheim, University of Heidelberg, 2013–509 N-MA & 2020–593 N).

#### Cytogenetic analysis

After cultivation of bone marrow (BM) cells for 24 h at least 20 metaphases were analyzed by G-banding techniques as previously described^10^. Karyotypes were interpreted according to the International System for Human Cytogenetic Nomenclature (ISCN 2020)^11^.

#### BCR::ABL1 allele burden

For quantitative follow-up of the *BCR::ABL1* allele burden (AB), we used either LightCycler (LC) RT-qPCR method or TaqMan RT-qPCR technology, as previously described^12,13^.

#### JAK2 V617F variant allele fraction

For quantitative follow-up of the *JAK2* V617F variant allele fraction (VAF), we used QuantStudioTM three-dimensional (3D) digital PCR (dPCR) System (ThermoFisher Scientific, Waltham, MA, USA). Per sample, a 15 µL reaction was prepared. The volume included 7.1 µL of 10 ng/µL DNA, 7.5 µL of QuantStudioTM 3D Digital PCR Master Mix v2 (ThermoFisher Scientific, Waltham, MA, USA) and 0.4 µL of *JAK2* V617F specific TaqMan gene expression assay (ID: Hs000000038_rm, ThermoFisher ScientificWaltham, MA, USA). dPCR was performed using the following thermal cycling conditions: 96 °C for 10 min, (56 °C for 2 min, 98 °C for 30 s (x39 cycles)) and 56 °C for 2 min.

#### Next generation sequencing

Next generation sequencing (NGS) of a myeloid gene panel of 18 frequently mutated genes in MPN was performed through library preparation by the Access Array Technology (Fluidigm, San Francisco, CA) and sequencing on the MiSeq Instrument (Illumina, San Diego, CA). Gene mutations were annotated compared to the reference sequence of the Ensembl Transcript ID (Ensembl release 85, July 2016).

#### Colony-forming unit granulocyte–macrophage (CFU-GM)

1 × 10^5^ mononuclear cells (MNC) from peripheral blood (PB) were seeded in 1 ml 0.9% methylcellulose supplemented with 30% fetal bovine serum albumin (FBS), 1% bovine serum (BS) albumin, 0.1 M 2-mercaptoethanol and recombinant human GM-CSF (100 ng/ml; MethoCult, StemCell Technologies, Cologne, Germany). Per patient, we prepared 5 to 10 Petri dishes (35 mm diameter, 1 ml MethoCult each) and incubated the cells at 37 °C in a humidified atmosphere with 5% CO_2_ for 10 to 14 days. Single-cell-derived colony-forming units granulocyte–macrophage (CFU-GM, 100–300 cells per colony) were detached from the dishes and diluted in 100 µl phosphate-buffered saline (PBS)^14^.

#### Detection of BCR::ABL1 in CFU-GM

For detection of *BCR::ABL1* in CFU-GM, we used fluorescence in-situ hybridization (FISH) analysis. After colony collection from MethoCult in PBS, the colonies were separated in a ratio of 1:5. The cell suspension (80µL) was washed two-times with PBS before cytospinning onto teflon-coated slides. Slides were dripped with methanol and incubated for 30 min. For staining of *BCR-ABL1*^pos^ cells, we used XL BCR/ABL 1 plus (MetaSystems, Altlussheim, Germany) according to the manufacturer’s instructions^15^.

#### Detection of JAK2 V617F in CFU-GM

For detection of *JAK2* V617F in CFU-GM, DNA was extracted using whole-genome amplification (REPLI-g Mini Kit, Qiagen, Hilden, Germany) according to the manufacturer’s instructions. Qualitative detection of *JAK2* V617F was performed as previously described. Colonies were only considered evaluable when both analyses (PCR for *JAK2* V617F and FISH for *BCR::ABL1*) delivered evaluable results.

### Statistical analysis

All statistical analyses considered clinical, laboratory, histological and molecular parameters obtained at the time of diagnosis/first referral of MPN-CML. OS analysis was considered from the date of diagnosis to date of death or last visit. OS probabilities were estimated using the Kaplan-Meier method. The correlation between variables was investigated using two-sided Mann-Whitney U-test (t-approximation) and was considered statistically significant with a *p* < 0.05.

## Results

### Clinical characteristics and laboratory findings

Between 2009 and 2023, we identified 10 patients with concomitant presence of *JAK2* V617F and *BCR::ABL1* who were tested for *JAK2* V617F and *BCR::ABL1* at diagnosis or during follow-up in approximately 9,000 patients. Patients were either concurrently positive for *JAK2* V617F and *BCR::ABL1* at initial diagnosis (5/10, 50%; patients #1 to #5, cohort A) or *BCR::ABL1* emerged in a preexisting *JAK2* V617F^pos^ MPN (5/10, 50%, patients #6 to #10, cohort B). Individual patient characteristics including clinical, morphological, laboratory, cytogenetic/genetic data and treatment/outcome data are presented in Tables 1 and 2.

**Table 1:**
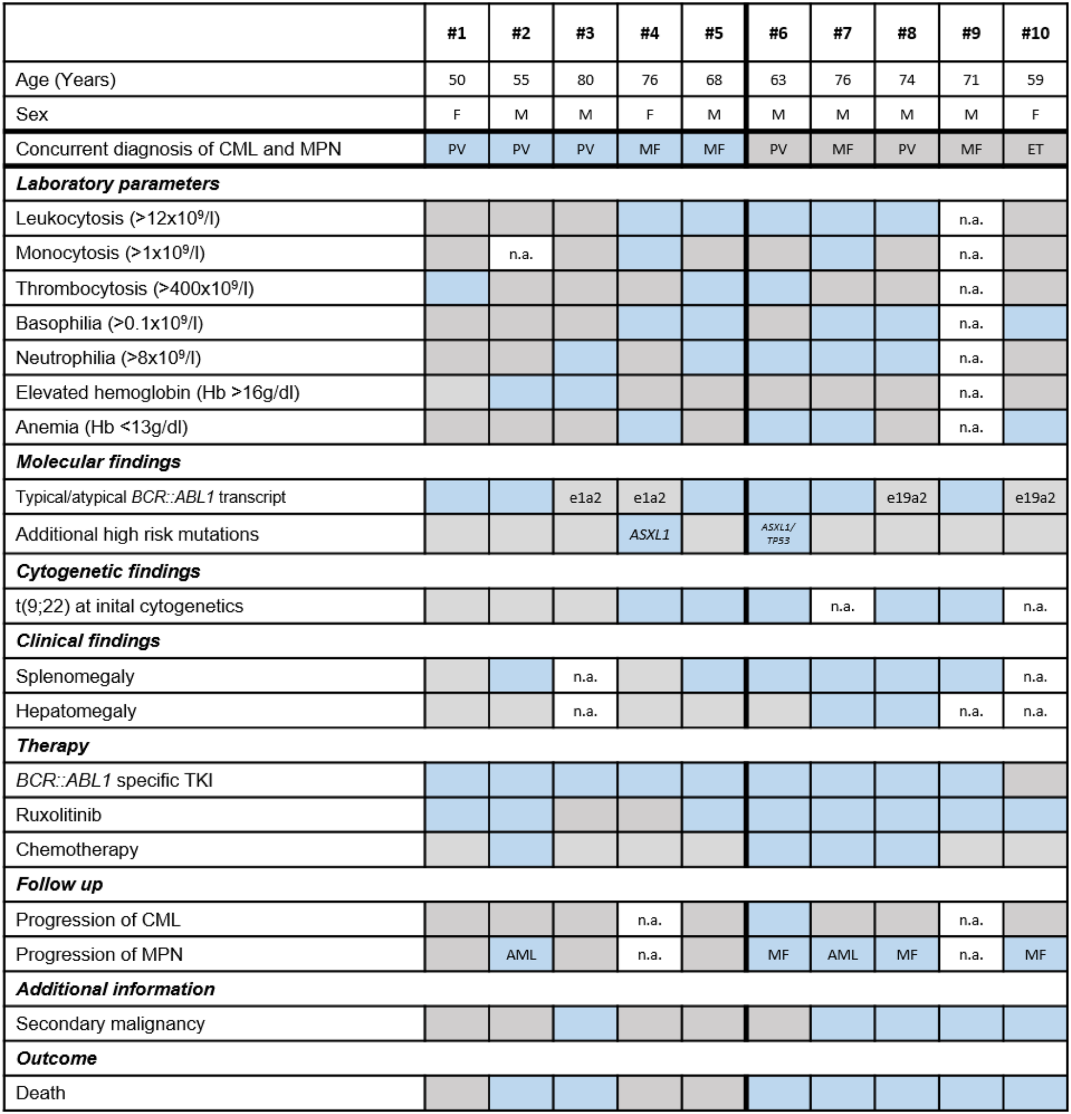
Patients characteristics for cohort A (#1-5, simultaneous MPN-CML) and B (#6-10, MPN first). F, female; M, male; n.a., not available; CML, chronic myeloid leukemia; MPN, myeloproliverative neoplasm; ET, essential thrombocythemia; PV, polycythemia vera; MF, myelofibrosis; AML, acute myeloid leukemia; TKI, tyrosine kinase inhibitor. All data are collected from the timepoint of CML-MPN diagnosis. Blue fields represent „yes“ and grey fields represent „no“.

**Table 2:** Laboratory parameters of *JAK2* V617F^pos^/*BCR::ABL*1^pos^ patients at diagnosis of MPN-CML.

Variables	Cohort A	Cohort B	*P*-value
**Number of patients**, ***n***	**5**	**5**	--
Leukocytes, x10^9^/L; median (range)	11.1 (8.5–56.7)	23.5 (4.1-127.5)	n.s.
Neutrophils, %, median (range)	63 (9–86)	54 (8–92)	n.s.
Monocytes, x10^9^/L; median (range)	0.6 (0.4–5.4)	0.8 (0.0-2.4)	n.s.
Eosinophils, x10^9^/L; median (range)	0.4 (0.1–1.2)	0.1 (0.0-0.4)	n.s.
Basophils, x10^9^/L; median (range)	0.1 (0.1–4.2)	0.1 (0.0-6.5)	n.s.
Hemoglobin, g/dL; median (range)	15.1 (12.9–18.9)	10.8 (8.9–13.7)	0.013
Hematocrit, %; median (range)	45 (40–63)	32 (28–44)	n.s.
Platelets, x10^9^/L; median (range)	384 (106–957)	237 (109-1,161)	n.s.

Cohort A: simultaneous diagnosis; Cohort B: MPN first; Abbreviation: n.s., not significant.

### Cohort A: concurrent diagnosis of *JAK2* V617F and *BCR::ABL1*

#### Baseline characteristics

The median age at initial diagnosis was 68 (range 50–80) years. The histopathologic phenotype of BM was available in 4/5 patients (#1, ET; #2, chronic myelomonocytic leukemia [CMML] with fibrosis; #3, myeloproliferative neoplasm - unclassified [MPN-U]; #5, MF). Of note, BM morphology was not indicative of CML in any patient at diagnosis and neither in 3/5 patients with repetitive BM biopsies. Initial cytogenetic and/or FISH analysis revealed a typical t(9;22)(q34;q11) in 2/5 patients (#4, #5) and an atypical karyotype in 3/5 patients (#1: 46,XX[31]; #2: 46,XY,−5,+mar[2]/46,XY[15]; #3: 46,XY, der(11)t(9;11)(p21;p15)[7]/46,XY[8]). Patient #2 developed a complex aberrant karyotype (51,XY,−6,+8,+13,+21,+21,+22,+mar[20]) within 10 months. *BCR::ABL1* fusion transcripts were e13a2/e14a2 in 3/5 patients (#1, #2 and #5) and e1a2 in 2/5 patients (#3 and #4). *BCR::ABL1*^IS^ at diagnosis was 0.5% (#1), 2% (#2) and 0.6% (#5) and *BCR::ABL1/GUSB* quotient was 0.6% in patient #3 and 36% in patient #4 (both patients had atypical transcripts). Given the low *BCR::ABL1*^IS^ allele burden, presence of a *BCR::ABL1* CHIP cannot be ruled out completely in pts #3 and #5. Additional somatic mutations were detected in 2 patients (#3, *TET2* p.Phe1309Leu, 2.6% VAF; #4, *ASXL1* p.Trp898*, 6% VAF and p.Glu635Argfs*15, 33% VAF).

#### Treatment and outcome

**Fig. 1:**
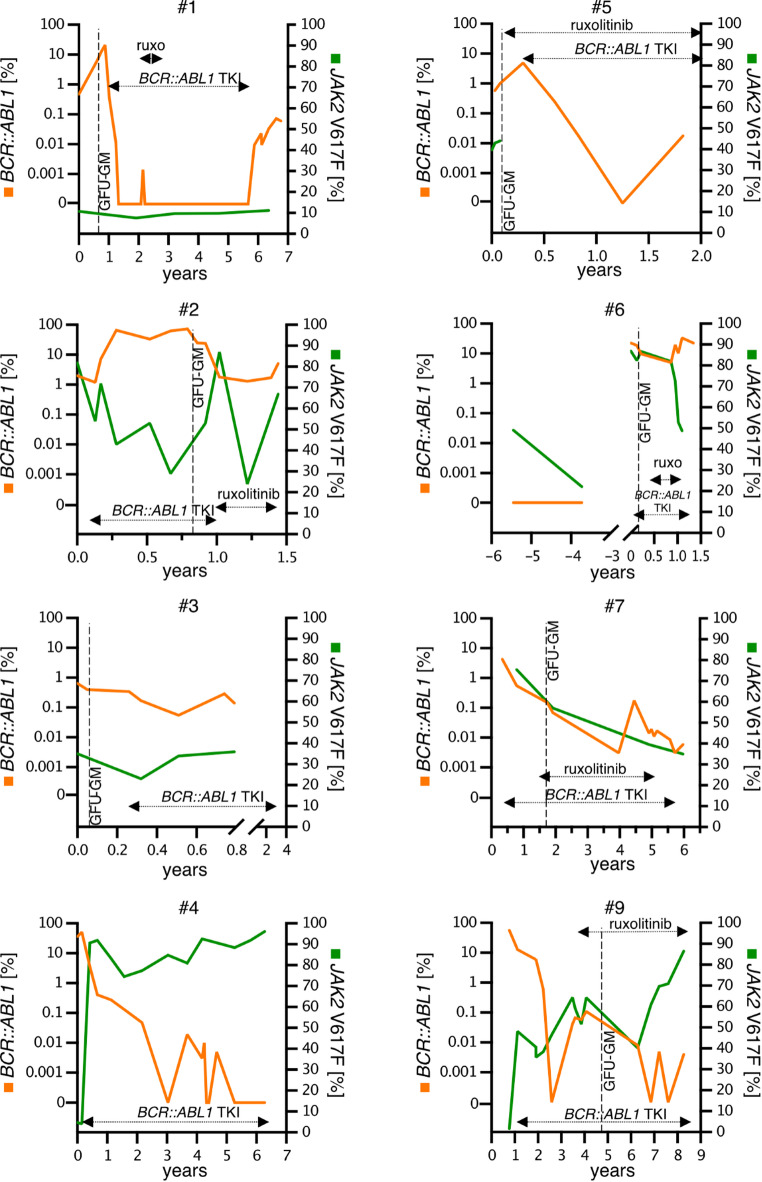
Individual courses of 8/10 patients regarding mutation allele frequencies, therapy and time point of CFU-GM. For individual courses of patients #8 and #10 see Fig. 3. Abbreviations: TKI, tyrosine kinase inhibitor; ruxo, ruxolitinib; CFU-GM, colony-forming unit granulocyte–macrophage assay.

All 5 patients were initially treated with an ABL1-inhibitor (imatinib, *n* = 3; nilotinib, *n* = 1; dasatinib; *n* = 1). Two patients were switched to an alternative ABL1-TKI (dasatinib, *n* = 2) because of lack of response (*n* = 1) or adverse events (AE, *n* = 1). A relevant molecular response (MMR in e13a2/e14a2 or ≥ 3-log reduction in e1a2) was achieved in 2/3 and 2/2 patients, respectively (imatinib, *n* = 1; dasatinib, *n* = 2; nilotinib, *n* = 1), which was durable in 3/4 patients for median 49 (range 12–63) months until last visit. In patient #2, 2nd line treatment with dasatinib resulted in a sustained remission of the *BCR::ABL1* fusion (*BCR::ABL1*^IS^ < 2%) and was stopped after 6 months due to progression into MPN blast phase with a complex aberrant karyotype and massive increase of the *JAK2* V617F VAF (66%). Three of five patients (#1, 2 and 5) were additionally treated with ruxolitinib over median 4 months (range 2–21, Fig. 1). In two patients, treatment was only short due to intolerance (#1, exanthema) or death as a result of treatment-resistant MPN blast phase (#2). In patient #5, splenomegaly and symptoms improved, but thrombocytosis and *JAK2* V617F VAF remained unaffected on 30 mg total daily dose of ruxolitinib, which was therefore increased to 40 mg daily. In none of the patients, either TKI dosage had to be adjusted due to cytopenia while on combination treatment. Addition of further cytoreductive treatment was necessary in two of the five patients only (#1: hydroxyurea in lieu of ruxolitinib which was stopped due to intolerance and #2 induction chemotherapy due to sAML progression. The median follow-up time between diagnosis and death or last contact was 4 (range 1–8) years with a median OS of 8.2 years. Two patients died due to progression (*n* = 1) or comorbidity (liver cell carcinoma, *n* = 1).

#### CFU-GM

**Fig. 2:**
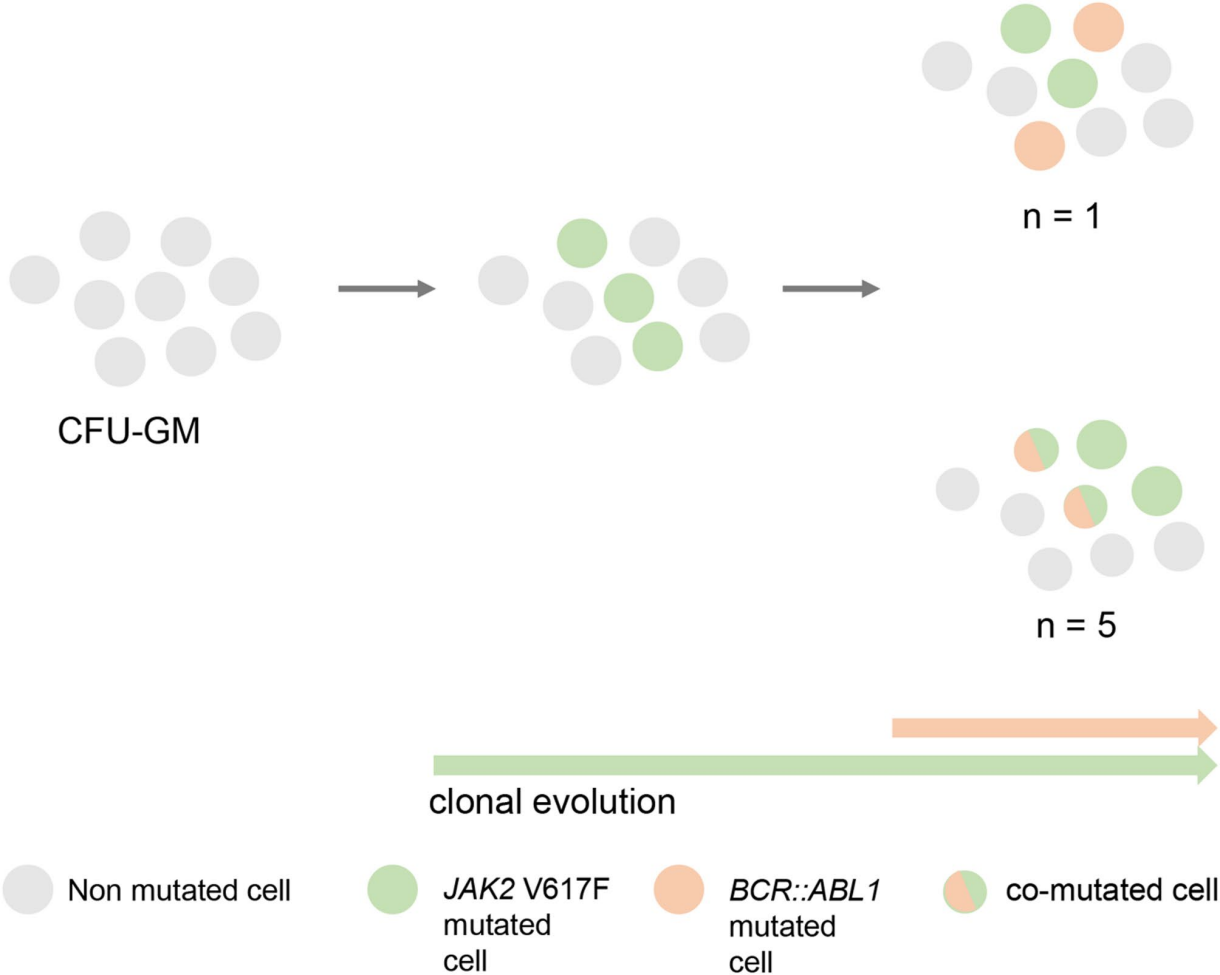
Molecular architecture and hierarchy of *JAK2* V617F and *BCR::ABL1* positive patients. Acquisition of *JAK2* V617F occurred as an early event in mutational hierarchy. Subsequently, two scenarios were possible: either occurrence of *BCR::ABL1* as second event in the already *JAK2* V617F positive clone or acquisition of an independent *BCR::ABL1* positive clone.

CFU-GM were available in 4/5 patients (#1, 2, 3, 5) either prior to (#1, 3 and 5) or on (#2, 10 months) treatment with an ABL1-TKI. At this time point, the *BCR::ABL1* was 6.5%, 25%, 0.4%, 1% and the *JAK2* V617F VAF was 10%, 40%, 30% and 44%, respectively. In patient #1, 6/15 informative colonies were *JAK2* V617F^pos^, 1/15 colonies were *JAK2* V617F^pos^/*BCR::ABL1*^pos^ and 0/15 *BCR::ABL1*^pos^. In patient #2, the numbers were 12/13, 1/13 and 0/13, respectively. These numbers indicate that, despite concurrent diagnosis of both mutations, *BCR::ABL1* most likely emerged in a *JAK2* V617F^pos^ clone (Fig. 2). In patients #3 and #5, the numbers of informative colonies were 18 and 14, respectively, with low numbers of positive CFU-GM for either mutation or even negative results (*JAK2* V617F^pos^, #3, 2/18; #5, 1/14; *BCR::ABL1*^pos^, #3, 0/18; #5, 2/14). In these two, yet untreated patients, none of the colonies tested positive for both mutations. As consequence, no definite conclusions can be drawn for patient #3 upon potential presence of two independent diseases versus the emergence of *BCR::ABL1* in a preexisting *JAK2* V617F^pos^ clone or vice versa. However, due to presence of *JAK2* V617F^pos^ colonies only, the earlier occurrence of a founding *JAK2* V617F^pos^ clone seems the more likely scenario. On the other hand, the complete absence of *BCR::ABL1* in CFU-GM may be associated with the simultaneously low *BCR::ABL1* (AB: 0.4%, e1a2; Supplementary Fig. 1). Given the presence of *BCR::ABL1*^pos^ and *JAK2* V617F^pos^ clones in patient #5, the presence of two separated clones is more likely.

### Cohort B: emergence of *BCR::ABL1* in a pre-existing *JAK2* V617F^pos^ MPN

#### Baseline characteristics

The median age was 60 (range 38–69) years at diagnosis of MPN and 71 (range 59–76) years at first appearance of *BCR::ABL1*. The median time between diagnosis of *JAK2* V617F^pos^ MPN and *BCR::ABL1*^pos^ CML was 14 (range 2–20) years. Initial diagnoses included PV, *n* = 2, ET, *n* = 1, MF, *n* = 2. At acquisition of *BCR::ABL1*, the histopathologic phenotype was re-evaluated in 2/5 patients (#6, PV; #7, CML with MF grade II). In patient #6, a follow-up biopsy after 14 months revealed MF grade II. Cytogenetic analysis and FISH revealed a t(9;22) in 3 informative patients (#6, #8, #9), with an additional del(20q) and a + 9 in patient #9. *BCR::ABL1* transcripts were e13a2/e14a2 (*n* = 3) and e19a2 (*n* = 2; #8 and #10). *BCR::ABL1*^IS^ at diagnosis was 17.5% (#6), 18.5% (#7) and 10.7% (#9) and *BCR::ABL1/GUSB* quotient was 21.5% in patient #8 and 0.07% in patient #10 (both patients had atypical *BCR::ABL1* transcripts). Additional somatic mutations were identified in 3 patients (#6, *ASXL1* p.Gly646Trpfs*12, 32% VAF, *TET2* p.Tyr559Leufs*8, 40% VAF, *TP53* p.Leu257Pro, 6% VAF; #7, *TET2* p.Gly1152Arg, 42% VAF; #8, *TET2* p.Gln417*, 41% VAF).

#### Treatment and outcome

After acquisition of *BCR::ABL1*, 4 of 5 patients were treated with imatinib and 3 of 4 patients were switched to nilotinib (#7; #9), dasatinib (#6, #7, #9) and bosutinib (#7) because of lack of response (*n* = 2) or AEs (*n* = 1). Three of 4 patients finally achieved a deep remission (MMR, ≤ 0.1% in e13a2/e14a2 or > 3-log reduction in e19a2 on imatinib (*n* = 1) or nilotinib (*n* = 2). The MMR was durable in 3/3 patients for median 53 (range 41–68) months until last visit. The median observation time between MPN-CML diagnosis and death or last contact was 6 (range 1–9) years. The median OS was 21.5 and 7.7 years after identification of *JAK2* V617F and emergence of *BCR::ABL1*, respectively.

**Fig. 3:**
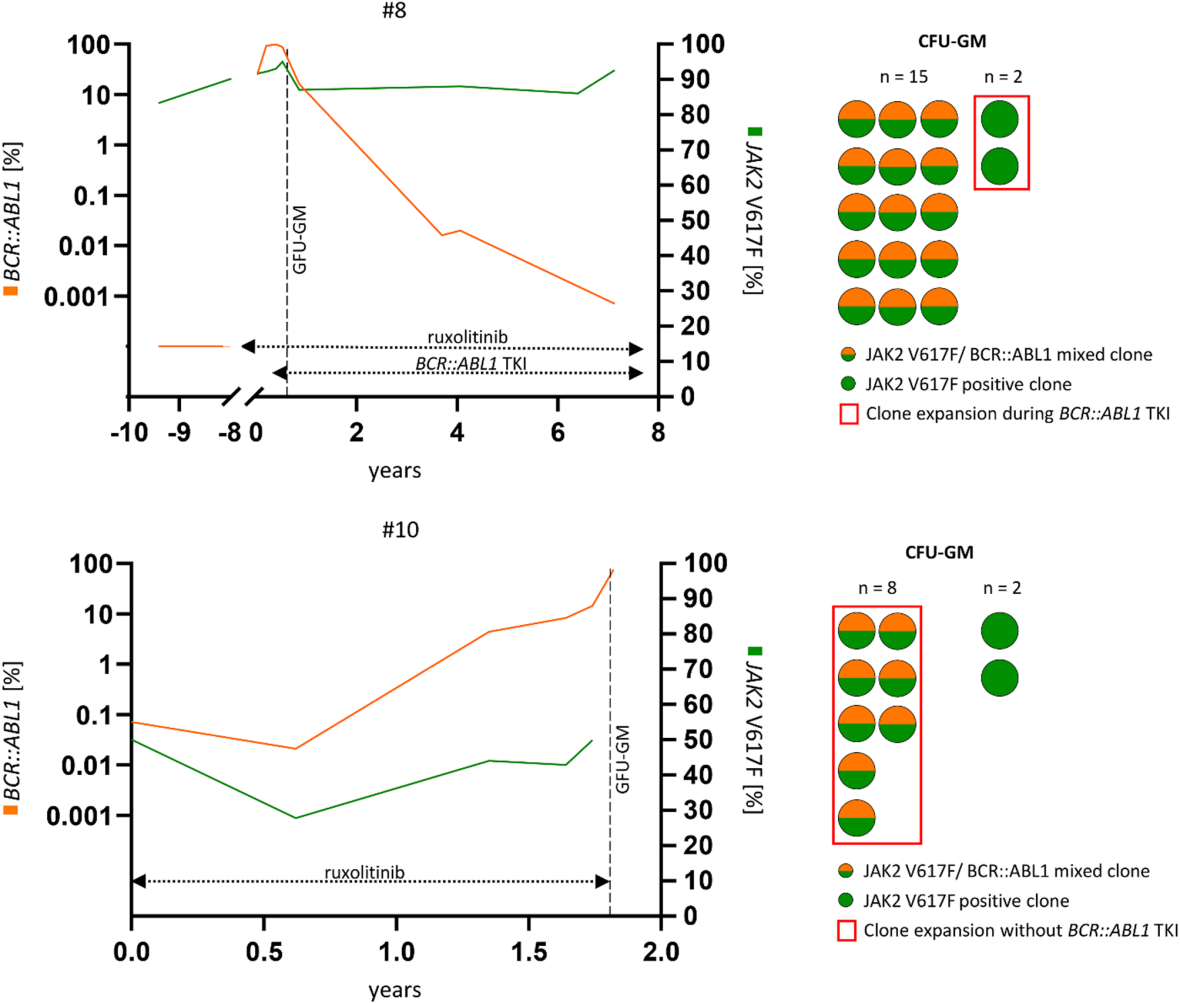
Individual courses of *JAK2* V617F and *BCR::ABL1* mutation allele burden with corresponding single cell clonality analysis. Patient #8 represents *JAK2* V617F^pos^/*BCR::ABL1*^pos^ cases in which the course of allele burden do not match the single cell analysis data. This patient expresses a mixed *JAK2* V617F^pos^/*BCR::ABL1*^pos^ clone with clonal expansion of the *JAK2* V617F^pos^ clone during tyrosine kinase treatment. Patient #10 represents *JAK2* V617F^pos^/*BCR::ABL1*^pos^ cases in which the course of allele burden matches the single cell analysis data. This patient expresses a mixed *JAK2* V617F^pos^/*BCR::ABL1*^pos^ clone with clonal expansion of the *JAK2 V617F*^pos^/*BCR::ABL1*^pos^ clone without tyrosine kinase inhibitor treatment. Abbreviations: CML, chronic myeloid leukemia; MPN, myeloproliferative neoplasm; CFU-GM, colony-forming unit - granulocyte, monocyte.

**Fig. 4:**
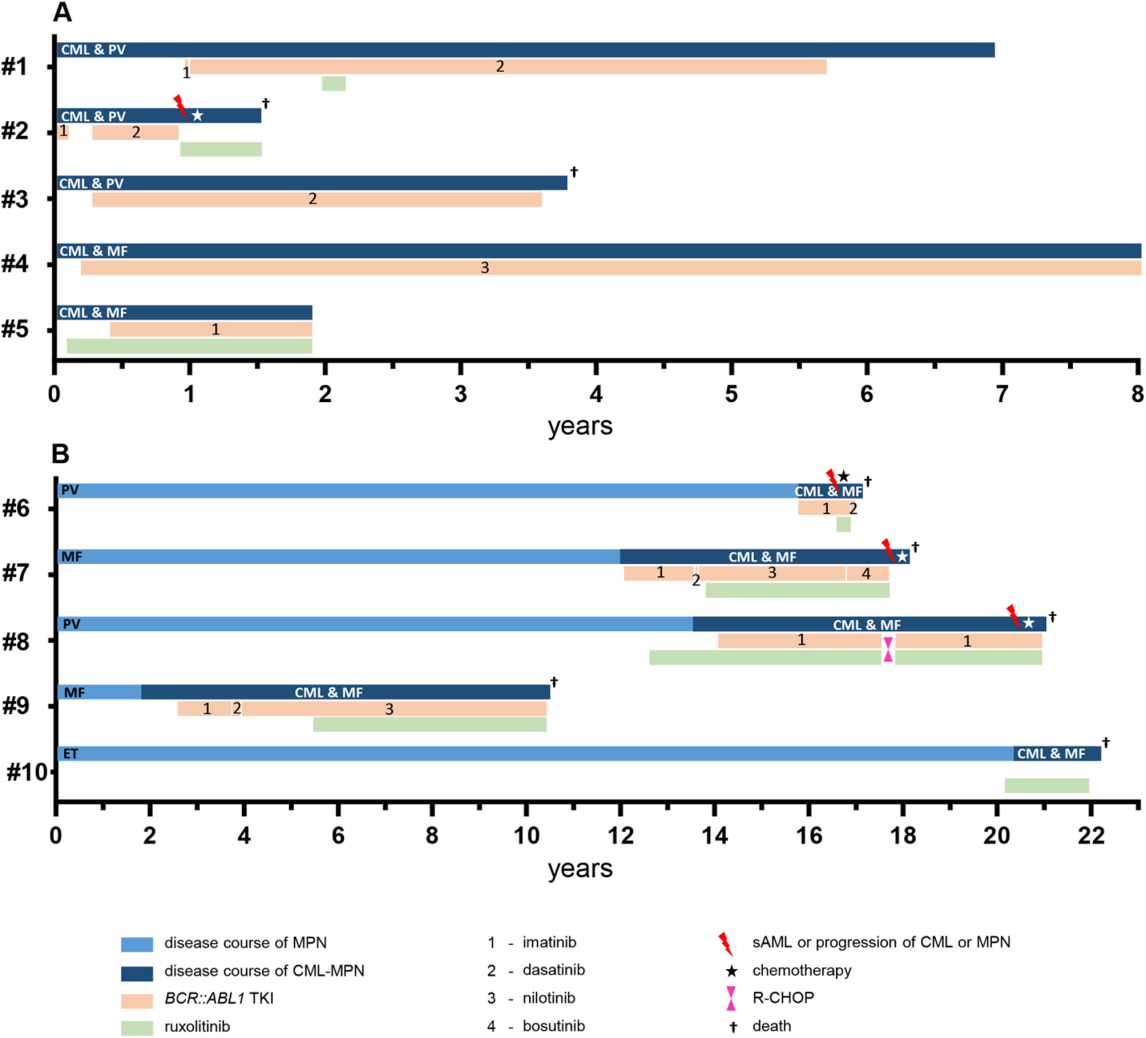
Individual clinical and treatment course of *JAK2* V617F and *BCR::ABL1* positive patients. (**A**) Patients with simultaneous diagnoses of CML and MPN (cohort **A**) and (**B**) Patients with consecutive diagnoses of CML and MPN (cohort **B**). Abbreviations: CML, chronic myeloid leukemia; PV, polycythemia vera; MF, myelofibrosis; ET, essential thrombocythemia; MPN, myeloproliferative neoplasm; AML, acute myeloid leukemia.

Hydroxyurea for cytoreduction was applied as first line treatment before ruxolitinib in 3 patients but eventually switched to ruxolitinib resulting in all 5 patients from cohort B being treated with ruxolitinib. JAK-inhibitor treatment showed a response of splenomegaly (4/5) and symptoms (5/5), whereas the *JAK2* V617F VAF remained unchanged in most patients (#6, #8, #10; Fig. 3). An increase (#9, maximum VAF 90%, + 30%) or decrease (#7, minimum VAF 30%, −27%) of the *JAK2* VAF was only seen in one patient, respectively (Fig. 1). Neither the ruxolitinib dosage nor the ABL1 TKI dosage had to be adjusted due to cytopenias in all four patients with combination treatment. Anemia grad 1 was present in patients #6 and #9 but did not deteriorate on treatment. Interestingly, in patient #7, MMR was only reached after initiation of the combination treatment. Four patients (#7,#8,#9,#10) developed secondary solid cancers (non-melanoma skin cancer, *n* = 3; sarcoma, *n* = 1, penile carcinoma, *n* = 1; colon carcinoma, *n* = 1, high grade b cell lymphoma, *n* = 1 and breast cancer, *n* = 1) with a remarkable clustering in patient #8: this patient had a history of a high-grade B-cell lymphoma, a colon carcinoma, a penile carcinoma and several dozen basaliomas and spindle cell carcinomas of the skin, the latter probably also related to long-term treatment with hydroxyurea. Median 18.5 years after first identification of *JAK2* V617F (median age 61 years) and median 6 years after identification of the concurrent presence of both mutations (median age 71 years), all five patients have died. Causes of death included comorbidity (*n* = 3) and progression (*n* = 2) (Fig. 4).

#### CFU-GM

CFU-GM were available in all 5 patients prior to (*n* = 2, patient #9, 10) or on treatment with an ABL1-inhibitor (*n* = 3, patient #6, 7, 8). At this time point, the *BCR::ABL1* AB in PB was 19%, 0.15%, 88%, 0.03% and 75%, and the *JAK2* V617F VAF was 82%, 60%, 95%, 50% and 50%, respectively. In patients #8–10, informative colonies were *JAK2* V617F^pos^/*BCR::ABL1*^pos^ in 15/17, 4/20 and 8/10 CFU-GM, respectively. *JAK2* V617F^pos^ CFU-GM were identified in 2/17, 9/20 and 2/10 colonies, respectively, while no CFU-GM were exclusively *BCR::ABL1*^pos^. In line with the patient’s history, these findings suggest the later acquisition of *BCR::ABL1* in *JAK2* V617F^pos^ CFU-GM. In patients #6 and #7, colonies were only *JAK2* V617F^pos^ (14/14 and 8/16). Concordantly, patient #7 had a relatively low *BCR::ABL1*^IS^ of 0.15% after 3 weeks on nilotinib (previously imatinib and dasatinib) with a concomitant high *JAK2* V617F VAF of 60%. In patient #6, the negativity of CFU-GM for *BCR::ABL1* despite a relatively high *BCR::ABL1*^IS^ of 19% could be due to the fact that in this case the lymphoid cells (and not myeloid cells covered with our assay) were positive for *BCR::ABL1* (Supplementary Fig. 1).

## Discussion

In chronic myeloid neoplasms, the concurrent presence of two driver mutations, which individually often leads to heterogeneous clinical disease phenotypes, is rare. The coincidence of *JAK2* V617F^pos^ MPN and *BCR::ABL1*^pos^ CML was estimated at 0.04% and 0.2% per year, respectively^16,17^. We here report on the combination of clinical, morphological and genetic characteristics of 10 *JAK2* V617F^pos^/*BCR::ABL1*^pos^ patients including molecular data on CFU-GM in 9/10 patients and the response on concurrent or sequential treatment with ABL1- and JAK-TKI which clearly distinguishes our work from previously published case reports.

The two most recently published reviews on altogether 63 case reports or case series totaling 142 cases reported (i) concurrent diagnosis of CML/MPN in 49% and 20%, (ii) CML emerging in MPN in 28% and 62%, and (iii) MPN emerging in CML in 23% and 18% of patients, respectively^9,18^. In contrast, we only observed concurrently diagnosed CML/MPN and CML emerging in MPN, but no case of an MPN emerging in CML. Interestingly, updated analyses of patients with presumed pre-existing *BCR::ABL1*^pos^ CML revealed positivity for *JAK2* V617F in the initial sample in 38%. In the remaining cases, *JAK2* V617F was not tested for in the baseline sample (35%) or the selected assays may not have been sensitive enough for adequate detection of *JAK2* V617F (27%; Table 3). In such cases, *JAK2* V617F could have been a mutation of clonal hematopoiesis of indeterminate potential (CHIP) whose VAF was below the detection limit of conventional sequencing or NGS assays for many years prior to diagnosis of an MPN.

**Table 3:** Retrospective evaluation of first *JAK2* V617F occurrence in published case reports/small series of patients with initial diagnosis of ph+^+^ CML followed by ph+^−^ MPN.

First Author	Year	Patients [*n*]	Retrospective JAK2 V617F analysis at time of CML diagnosis	JAK2 V617F Allele burden (%)	Limitations
Krämer^34^	2007	1	***JAK2*** **V617**^**pos**^	~ 40	
Inami^35^	2007	1	***JAK2*** **V617**^**pos**^	~ 20	
Hussein^36^	2007	1	***JAK2*** **V617**^**pos**^	~ 5	
Kim^37^	2008	2	***JAK2*** **V617**^**pos**^	> 60	
Hussein^38^	2008	2	*JAK2* V617^neg^ (*n* = 1), n.d. (*n* = 1)		a
Gattenlohner^39^	2009	1	***JAK2*** **V617**^pos^	5	
Tefferi^40^	2010	1	*JAK2* V617^neg^		b
Caocci^41^	2010	1	n.d.	n.d.	
Véronèse^42^	2010	1	***JAK2*** **V617**^pos^	0.5	
Hummel^43^	2012	1	n.d.	n.d.	
Lee^44^	2013	2	*JAK2* V617^neg^		c
Pastore^45^	2013	1	***JAK2*** **V617**^pos^	0.122	
Pagoni^46^	2014	1	*JAK2* V617^neg^		c
Martin-Cabrera^17^	2016	1	***JAK2*** **V617**^pos^	28	
Darling^47^	2017	1	**n.d.**	n.d.	
Boddu^48^	2018	2	***JAK2*** **V617** n.d. (*n* = 1)	0.7	
Soderquist^16^	2018	1	n.d.	n.d.	
De Roeck^49^	2018	1	n.d.	n.d.	
Sorà^50^	2021	1	n.k.	n.d.	
Zhao^51^	2022	1	*JAK2* V617^neg^		d
Tosoni^52^	2023	1	n.d.	n.d.	
Lapietra^53^	2023	1	*JAK2* V617^neg^		c

Ph, philadelphia chromosome; n.d., not done; n.k., not known, (a) Limit of detection (LOD) was 1%. (b) LOD was 0.5%. (c) LOD was not specified. In one patient, massive thrombocytosis was already present at month 3 after diagnosis of CML (d) Limit of detection unknown (either LOD by qualitative PCR [0.1%], semiquantitative PCR [1%] or NGS [5%].

In 5/9 patients from our series, the genetic profile of CFU-GM indicates later acquisition of *BCR::ABL1* in a pre-existing *JAK2* V617F^pos^ MPN. In 3 further patients with various levels of remission on different ABL1-inhibitors, only *JAK2* V617F^pos^ colonies were identified adding up to 8/9 (89%) patients in which CFU-GM analyses hint towards a pre-existing *JAK2* V617F^pos^ MPN. We only identified one patient with two *JAK2* V617F^neg^/*BCR::ABL1*^pos^ colonies potentially indicating acquisition of *JAK2* V617F in a pre-existing *BCR::ABL1*^pos^ CML or the scenario of two independent diseases but cannot rule out potential technical pitfalls because *JAK2* V617F VAF was in fact high. In the literature, CFU-GM and/or BFU-E in vitro clonality analyses were reported in 10 *JAK2* V617^pos^/*BCR::ABL1*^pos^ cases^19–27^. For example, in a patient with a *BCR::ABL1*^pos^ CML emerging in a pre-existing *JAK2* V617F MPN, analyses of pooled CFU-GM and BFU-E identified *BCR::ABL1* only in CFU-GM but not in BFU-E while *JAK2* V617F was identified in both compartments suggesting earlier acquisition of *JAK2* V617F at the stem cell level^20^. Similar to our data, 5/7 reports using CFU-GM and/or BFU-E described *BCR::ABL1* emerging in a pre-existing *JAK2* V617F^pos^ MPN^19,22–24,27^. In another report, all but one clone were either *JAK2* V617F^pos^ or *BCR::ABL1*^pos^ while the only remaining clone was positive for both mutations^25^. In summary, there is no single cell-based in vitro assay that clearly detected two clonally independent diseases and all but one indicated *BCR::ABL1* emerging in pre-existing *JAK2* V617F^pos^ MPN. Of interest, this data contrasts with our recent report on *JAK2* V617F and *KIT* D816V in patients with concurrent diagnosis of a MPN and systemic mastocytosis. CFU-GM analyses revealed absence of concurrently *JAK2* V617F^pos^/*KIT* D816V^pos^ colonies and discordant development of the VAF for both mutations during follow-up indicating disease evolution in two independent clones rather than monoclonal disease in > 60% of patients examined.

The concomitant presence of *JAK2* V617F and the high rate of atypical *BCR::ABL1* fusion transcripts may indicate a different mechanism of formation of *BCR::ABL1* in the context of *JAK2* V617F. However, the relative overall frequency of achieving and duration of MMR did not differ from expected responses in “pure” CML^28^. The deep and durable *BCR::ABL1* responses show that the concurrent presence of the *JAK2* V617 mutation in the same clone is no reason for primary resistance. However, when interpreting these results, the small number of patients and the lack of internationally standardized and validated assays to monitor measurable residual disease in patients with atypical *BCR::ABL1* fusion transcripts must be taken into account^29^. Additional treatment with ruxolitinib was initiated in 8/10 patients. Treatment was rapidly discontinued in 2 patients due to intolerance or disease progression. Concurrent treatment with variable ABL1-TKI and ruxolitinib was administered in 6/10 patients. All 6 patients experienced responses of splenomegaly and/or symptoms but leukocytosis and/or thrombocytosis required addition of hydroxyurea in 3/6 patients whereas dosage reductions due to hematotoxicity was not necessary in any of the patients treated with an ABL-TKI and ruxolitinib, This contrasts with the scarce literature on combination treatment, where anemia and/or thrombocytopenia during combination treatment were frequently observed.

The subtle morphologic diagnosis and differentiation between coexisting *JAK2* V617F^pos^ MPN and *BCR::ABL1*^pos^ CML is complicated by the fact that left-shifted leukocytosis, thrombocytosis, BM hypercellularity and myelofibrosis can variably occur in both entities. While basophilia and eosinophilia, particularly in combination, are more typical for *BCR::ABL1*^pos^ CML, elevated hemoglobin/hematocrit and the presence of normoblasts in PB are more typical for *JAK2* V617F^pos^ MPN. In our cohort, the clinical MPN phenotype predominantly resembled PV, while (excessive) thrombocytosis was rare. Similar to the literature, patients with a simultaneous diagnosis of CML-MPN more frequently presented with only mild or moderate leukocytosis. In MPN patients receiving treatment, e.g. hydroxyurea, emerging CML variably presented with leukocytosis, basophilia and splenomegaly but not to the extent that would be expected in de novo CML. In contrast to the literature^16,18,30^, the histopathological features in our series were characterized by hypercellularity, atypical megakaryocytes and myelofibrosis (more primary in cohort A, more secondary in cohort B) in most patients, and a primary histopathological diagnosis of CML was not made in any of 8 patients studied.

The lack of clearly distinguishable cyto- and histomorphological features emphasizes the importance of profound genetic analyses through a combination of conventional cytogenetics, FISH and most importantly multiplex-PCR to identify *JAK2* V617F, a t(9;22) and/or typical and atypical *BCR::ABL1* fusion gene, respectively. In our series, only 5/9 patients had a typical t(9;22) and 4/10 patients had atypical *BCR::ABL1* fusion transcripts. Of note, other case reports described a frequency of e1a2 transcripts in *JAK2* V617F^*pos*^/*BCR::ABL1*^pos^ patients of approximately 15%,^16^ while e1a2 transcripts are identified in less than 2% of patients with regular *BCR::ABL1*^pos^ CML^31,32^. Overall, a *BCR::ABL1* fusion gene can therefore easily be missed at diagnosis or during progression in the absence of a typical t(9;22), in PCR analysis that only detects e13a2/e14a2 and in NGS panels that generally only identify mutations such as *JAK2* V617F, *CALR* or *MPL* but not fusion genes.

Additional somatic mutations are detected in approximately 35% of patients with CML and their presence indicates genomic instability and propensity for clonal evolution^33^. In *JAK2* V617F^*pos*^ MPN, the frequency of additional somatic mutations is related to the MPN subtype, with incidence rates highest in myelofibrosis. The overall frequency and relative distribution of additional somatic mutations in our cohort was not higher than in individual *BCR::ABL1*^pos^ and *JAK2* V617F^*pos*^ patients and it is difficult to attribute them to the CML clone or the MPN clone in most cases. For cohort B, however, due to the strong association of e.g. deletion 20q to *BCR::ABL1*^neg^ MPN, we think that the occurrence of karyotype alterations and mutation acquisition prior to CML diagnosis is more likely. Of note, apart from the identification of *TP53*/*ASXL1* and *ASXL1* alone in one patient each, no high molecular risk mutations according to recently established clinico-genetic scoring systems for myelofibrosis, e.g. MIPSS70+, were identified in the remaining 8 patients.

The combined evaluation of clonality analyses and clinical data is the strength of our work and clearly distinguishes our data from previously published cases. However, we acknowledge the limitations of our analysis in terms of a high number of non-evaluable colonies and the absence of confirming single cell genotyping assays for our cohort. We conclude that (i) the incidence of *JAK2* V617F^pos^/*BCR::ABL1*^pos^ myeloid neoplasms is low but if present, (ii) *BCR::ABL1* is usually acquired in a preexisting *JAK2* V617F^pos^ clone, therefore (iii) *JAK2* V617F^pos^ patients with progressive leukocytosis and/or (resistance to treatment) organomegaly should be tested for *BCR::ABL1*, (iv) despite an overrepresentation of atypical *BCR::ABL1* fusion transcripts on ABL1-TKI, similar to de novo CML high rates of MMR can be achieved and (v) simultaneous treatment with ruxolitinib is feasible, and an individualized drug response can be achieved with both specific treatment strategies.

## Electronic supplementary material

Below is the link to the electronic supplementary material.

Supplementary Material 1

## Data Availability

The original contributions presented in the study are included in the article/Supplementary Materials.
