# Supplementary figures and images for: Clinical, morphological and genetic characteristics of patients with concurrent presence of JAK2 V617F and BCR::ABL1
Source: Sci Rep. 2025 Jul 18;15:26046. doi: 10.1038/s41598-025-11096-6 (PMC12274285; doi:10.1038/s41598-025-11096-6)

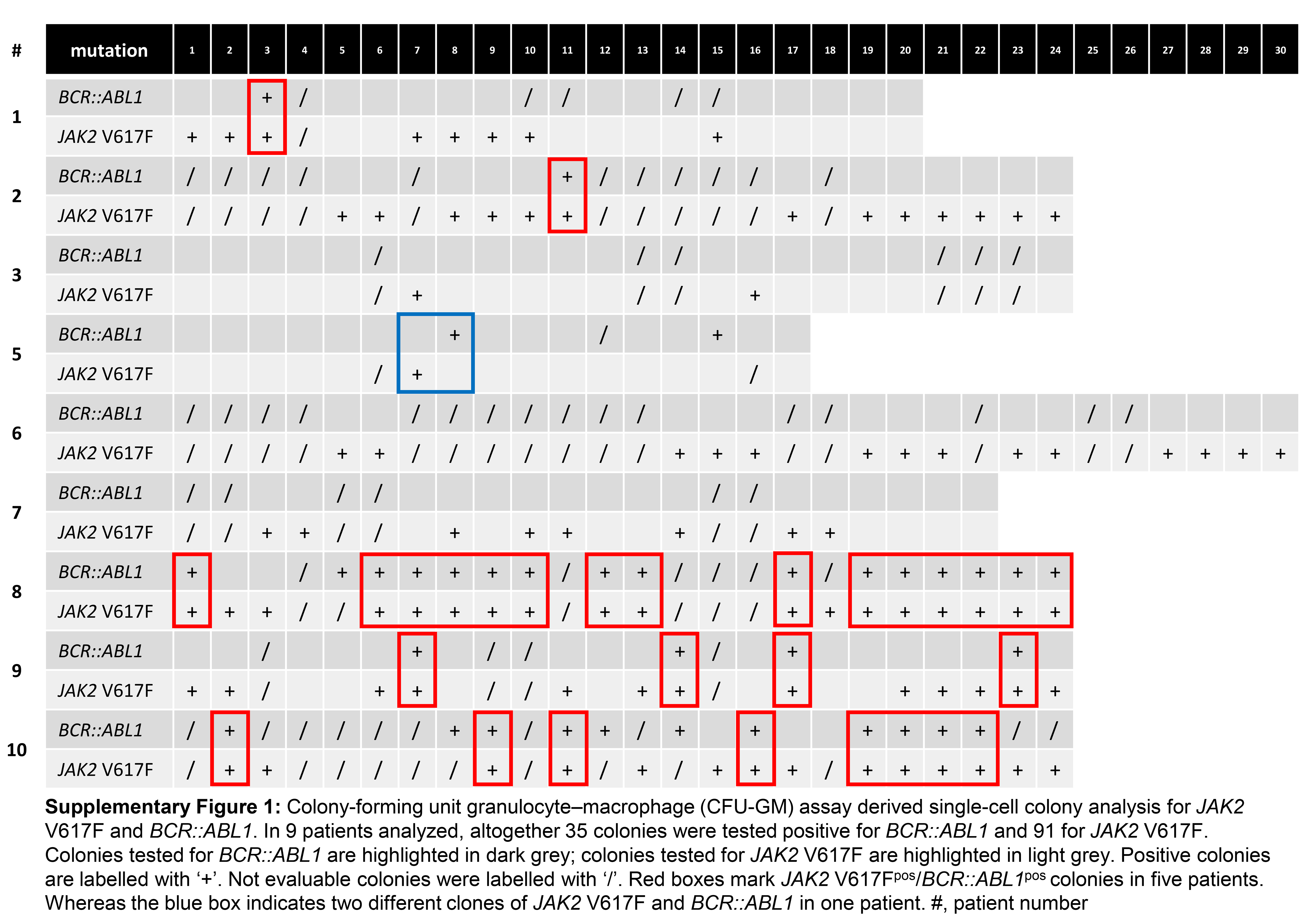

Supplement: Supplementary file 1 — Supplementary Material 1 [file 41598_2025_11096_MOESM1_ESM.tif]
